# Childhood Obesity, Hypothalamic Inflammation, and the Onset of Puberty: A Narrative Review

**DOI:** 10.3390/nu16111720

**Published:** 2024-05-31

**Authors:** Anastasia-Maria Tzounakou, Galateia Stathori, George Paltoglou, Georgios Valsamakis, George Mastorakos, Nikolaos F. Vlahos, Evangelia Charmandari

**Affiliations:** 1 Center for the Prevention and Management of Overweight and Obesity, Division of Endocrinology, Metabolism and Diabetes, First Department of Pediatrics, National and Kapodistrian University of Athens Medical School, ‘Aghia Sophia’ Children’s Hospital, 11527 Athens, Greece; anastasia.tzounakou@gmail.com (A.-M.T.); g.stathor@gmail.com (G.S.); 2 Diabetes Unit, Second Department of Pediatrics, National and Kapodistrian University of Athens Medical School, ‘P. & A. Kyriakou’ Children’s Hospital, 11527 Athens, Greece; gpaltoglou@gmail.com; 3 Second Department of Obstetrics and Gynecology, National and Kapodistrian University of Athens Medical School, ‘Aretaieion’ University Hospital, 11528 Athens, Greece; gedvalsamakis@yahoo.com (G.V.); mastorakg@gmail.com (G.M.); nfvlahos@gmail.com (N.F.V.); 4 Division of Endocrinology and Metabolism, Center of Clinical, Experimental Surgery and Translational Research, Biomedical Research Foundation of the Academy of Athens, 11527 Athens, Greece

**Keywords:** central precocious puberty, childhood obesity, hypothalamic inflammation, neuroinflammation

## Abstract

The onset of puberty, which is under the control of the hypothalamic–pituitary–gonadal (HPG) axis, is influenced by various factors, including obesity, which has been associated with the earlier onset of puberty. Obesity-induced hypothalamic inflammation may cause premature activation of gonadotropin-releasing hormone (GnRH) neurons, resulting in the development of precocious or early puberty. Mechanisms involving phoenixin action and hypothalamic microglial cells are implicated. Furthermore, obesity induces structural and cellular brain alterations, disrupting metabolic regulation. Imaging studies reveal neuroinflammatory changes in obese individuals, impacting pubertal timing. Magnetic resonance spectroscopy enables the assessment of the brain’s neurochemical composition by measuring key metabolites, highlighting potential pathways involved in neurological changes associated with obesity. In this article, we present evidence indicating a potential association among obesity, hypothalamic inflammation, and precocious puberty.

## 1. Introduction

Puberty is characterized by the development of primary and secondary sexual characteristics, ultimately resulting in the attainment of reproductive capacity [1]. The pubertal process arises as a result of the activation of the hypothalamic–pituitary–gonadal (HPG) axis, owing to the increased pulsatile secretion of gonadotropin-releasing hormone (GnRH), which leads to the increased secretion of gonadotropins [luteinizing hormone (LH) and follicle-stimulating hormone (FSH)] by the anterior pituitary and increased production of gonadal steroids (estrogen in females, testosterone in males) [2,3,4]. During pubertal development, physical changes occur in a predetermined sequence, yet the timing and pace of pubertal maturation differ significantly among individuals [4].

Precocious puberty (PP) is characterized by the development of secondary sexual characteristics before the age of 8 years in girls and 9 years in boys. PP is classified into central precocious puberty (CPP), when there is premature activation of the HPG axis, and peripheral precocious puberty (PPP), when there is increased production of gonadal steroid hormones, independently of gonadotropin secretion [5]. Most cases of CPP in girls are idiopathic; however, risk factors include a history of congenital or acquired central nervous system (CNS) anomalies, such as hypothalamic hamartoma, tumors, infections, or ischemia [6]. In addition, certain genetic syndromes, such as neurofibromatosis type 1, Prader–Willi syndrome, and Cowden syndrome, are associated with CPP [7]. In females, CPP is predominantly idiopathic, whereas in males it may arise as a consequence of underlying pathology [8]. Rapidly advancing puberty is indicated by advanced growth, uterine maturation, bone age, and elevated levels of LH. LH stands out as the most significant biochemical marker in diagnosing CPP. An early onset of menstruation is correlated with increased susceptibility to metabolic complications, such as obesity, type 2 diabetes, insulin resistance, and hypertension, as well as an elevated risk of cardiovascular ailments, including stroke and ischemic heart disease [7].

In the last few decades, there have been worldwide observations of a trend towards earlier pubertal onset in the general population, particularly among girls [9,10]. At the same time, the incidence of childhood obesity has markedly risen on a global scale over the last five decades. Recent findings indicate that the majority of obesity cases develop during the preschool period. Given that one in five obese preschool children are projected to transition into obese adults, this trend holds significant implications for public health. In 2019, the World Obesity Federation estimated that there would be 206 million children and adolescents aged 5–19 years living with obesity in 2025, and 254 million in 2030 [11,12]. It contributes to roughly 5% of global fatalities, predominantly due to cardiovascular disease. Obesity significantly affects both physical and psychological well-being, contributing to over 29 conditions previously seen mainly in adults. These include diabetes mellitus type 2 (T2DM), hypertension, metabolic syndrome, and various gynecologic disorders, such as early puberty and menstrual irregularities [13]. While the connection between obesity and CPP has been well established for some time, the specific mechanisms linking the two phenomena are still not fully understood.

Childhood obesity stems from various factors, but the leading cause is an imbalance between calorie consumption and energy expenditure, often associated with sedentary behaviors. Recognizing this reversible nature of the problem, extensive research has focused on understanding the factors behind this energy surplus and devising effective strategies for children and their families to adopt healthier lifestyles. Obesity entails a persistent, mild inflammation throughout the body, which impacts numerous organs. In human studies, there is evidence suggesting links between obesity and heightened gliosis across various brain regions, notably the hypothalamus, which plays a pivotal role in governing feeding behavior and metabolism [14].

The hypothalamus has a key role in controlling caloric intake and energy expenditure in the central control system of the brain. Research suggests that diet-induced inflammation primarily targets the mediobasal hypothalamus (MBH) in the early stages [15]. Based on studies using rodent models, signs of hypothalamic inflammation emerge within 1 to 3 days of consuming a high-fat diet. This suggests that hypothalamic inflammation occurs prior to the onset of weight gain [16]. Childhood obesity, a nutritional imbalance, may lead to early-onset puberty due to alterations in the production and/or release patterns of peptides associated with energy metabolism. Girls with obesity often experience an earlier onset of puberty compared to their age-matched, normal-body-mass-index (BMI) counterparts, and this has been associated with a higher BMI in adulthood [17]. Diet-induced hypothalamic inflammation may also cause premature activation of the GnRH neurons, resulting in the development of precocious or early puberty.

The objective of this review is twofold: firstly, to explore whether early or precocious puberty concurs with hypothalamic inflammation triggered by factors such as obesity, high-fat/high-glycemic-index diet, or other unidentified mechanisms, and secondly, to outline the brain magnetic resonance imaging (MRI) changes induced by obesity.

## 2. Obesity and Early or Precocious Puberty

Puberty, a multifaceted and coordinated biological process, displays considerable variation in onset among children due to influences from environmental, endocrine, and genetic factors. The overall prevalence of PP is estimated to be 1:5000 to 1:10,000 [18], with a female-to-male ratio of approximately 10:1, yet the exact pathophysiologic mechanism responsible for PP remains unknown [19]. Epidemiological data indicate that there is a direct correlation between early pubertal development and obesity [20,21]. Numerous research findings have underscored the association between overweight or obesity and the early onset of breast development and menarche in girls. The exact mechanisms responsible for the reactivation of the HPG axis remain unclear. The concept proposed by Frisch et al. in the early 1970s suggesting a specific BMI threshold required to trigger puberty initiation is no longer deemed credible [22]. Presently, researchers are focused on understanding how adipocytes influence pubertal activation, primarily through investigating the endocrine impact of adipose tissue. This involves studying adipokine secretion, insulin resistance, and the peripheral aromatase process as the most feasible theories underlying these mechanisms.

An increased BMI affects LH and FSH secretion by suppressing the HPG axis due to an abundance of circulating estrogen and androgen concentrations [23]. In addition, it has been widely recognized that body fat contributes not only to linear growth but also to bone maturation, independent of HPG axis activation. This discovery complicates the diagnosis of CPP in patients with obesity, as advanced bone maturation raises more concerns about true precocious puberty than the appearance of secondary sexual characteristics. In subjects with obesity and accelerated bone age, final adult height might be adversely affected owing to rapid linear growth in early childhood, offsetting premature epiphyseal closure during adolescence [24]. Unhealthy lifestyle daily behaviors and dietary patterns, along with factors such as inadequate sleep and high-fat diets (HFDs), have been suggested as contributors. More specifically, saturated fatty acids (SFAs) have been shown to modify the microbiota, affecting metabolic and inflammatory pathways [25]. Wang et al. showed that the gut microbiota and the SFAs it produces play a specific role in both the development and prevention of precocious puberty induced by obesity [26]. Furthermore, increased adiposity in prepubertal children may contribute to elevated aromatase activity, leading to an increased conversion of androgens to estrogens. Consequently, this could lead to an overexposure of tissues to estrogen during the prepubertal years [27].

## 3. Obesity, High-Fat Diet, and Hypothalamic Inflammation

**Figure 1:**
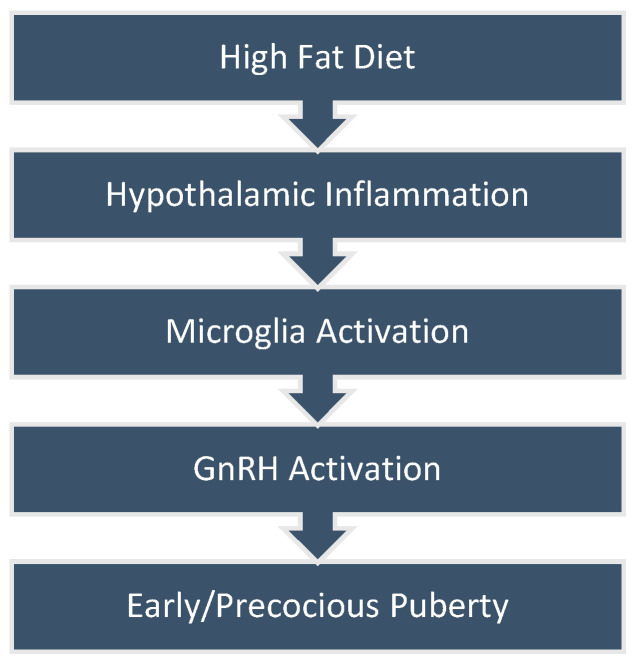
Diet-induced hypothalamic inflammation leading to precocious puberty.

Recent evidence suggests that a diet rich in fats triggers inflammation in the hypothalamus, disrupting energy balance and resulting in insulin resistance, glucose intolerance, and obesity. GnRH neurons are found in the medial preoptic area (POA) and in the arcuate (ARC) nucleus of the hypothalamus in humans [28]. Even though the exact processes initiating puberty still remain uncertain, the first identifiable biochemical change in this transition is the increased production of kisspeptin in the hypothalamus [29]. Although the stimulatory and inhibitory signals related to kisspeptin are not fully understood, research studies have demonstrated that elevated kisspeptin levels lead to increased release of GnRH. Consequently, the rise in kisspeptin is widely acknowledged as the pivotal event triggering the activation of the HPG axis during puberty [29,30]. Two mechanisms that have been associated with the premature activation of GnRH neurons are (i) the diet-induced Phoenixin action and (ii) hypothalamic microglia cells [23] (Figure 1).

### 3.1. Phoenixin

Phoenixin (PNX) is a newly identified brain peptide consisting of two active forms: phoenixin-14 and phoenixin-20. This newly discovered neuropeptide plays a significant role in various physiologic processes, such as reproduction, food intake, memory and anxiety, neuronal and microglial activity, and the regulation of inflammation [31]. Animal studies show that PNX stimulates reproductive functions by targeting neurons expressing both GnRH and kisspeptin 1 (Kiss1) within the HPG axis. PNX increases GnRH and GnRH receptor (GnRH-R) mRNA expression, as well as Kiss1 mRNA expression [32].

GPR173, PNX’s receptor, is expressed in the ARC and paraventricular nucleus (PVN), brain areas that are closely related to the regulation of food intake. Fatty acids stimulate PNX gene expression, indicating PNX’s involvement in sensing nutrition [33]. Mice on HFDs show increased hypothalamic expression of the PNX gene. Evidence suggests that an HFD might trigger the release of PNX, which acts on GnRH neurons, leading to GnRH activation [34]. Furthermore, PNX increases the sensitivity of the pituitary gland to the effects of GnRH. Specifically, the saturated free fatty acid palmitate, the monounsaturated fatty acid oleate, and polyunsaturated docosahexaenoic acid increase PNX gene expression in immortalized hypothalamic neuronal cell lines. This indicates a potential connection between increased fat levels in the hypothalamus and changes in the circulating levels of PNX observed in obesity [35].

### 3.2. Hypothalamic Microglial Cells

Microglia cells play an important role in PP. Microglial cells interact with GnRH cells, and this cellular communication is facilitated by prostaglandins [36]. Persistent exposure to an HFD can cause morphologic and physiologic alterations of microglia, leading to chronic inflammation [15]. Hypothalamic microglial cells are sensitive to fatty acids, and studies show that these cells undergo an inflammatory activation in mice fed with a diet rich in SFAs [37]. Additionally, Brain-Derived Neurotrophic Factor (BDNF) triggers the activation of astrocytes and microglia, resulting in the release of tumor necrosis factor-alpha (TNF-α) and interleukin-1 beta (IL-1β), thereby contributing to the exacerbation of neuroinflammation [38]. Bartosz et al. showed that in sheep treated with exogenous BDNF, increased GnRH mRNA expression was observed in the preoptic area (POA), along with increased mRNA expressions of kisspeptin in the MBH [39]. These findings suggest that BDNF might play a role in controlling the activity of the gonadotrophic axis.

Various metabolic signals regulate the HPG axis, with the interaction between leptin and kisspeptin being the most extensively studied. Leptin, an appetite-suppressing hormone, serves as a long-term regulator of body mass, dampening the activity of hunger-related neurons. When leptin binds to its receptor, it triggers the kisspeptin pathway, signaling to the hypothalamic GnRH pulse generator that energy reserves are sufficient for fertility. Although essential, this process alone does not fulfill the criteria for sexual development. Studies have shown that leptin levels increase before puberty onset in girls, with leptin peaks preceding those of gonadotropins.

Adipokines are active molecules released by adipose tissue, contributing to multiple physiological processes like energy metabolism, appetite control, insulin sensitivity, and inflammation regulation. Dysfunctions in adipose tissue, as triggered by a high-fat diet, can disrupt these functions by altering adipokine secretion. Adipokines have been linked to insulin resistance, a characteristic feature of obesity. In obese children, there is a reduction in adiponectin levels, which plays a role in increasing insulin sensitivity [23].

## 4. Brain MRI Findings in Obesity

**Figure 2:**
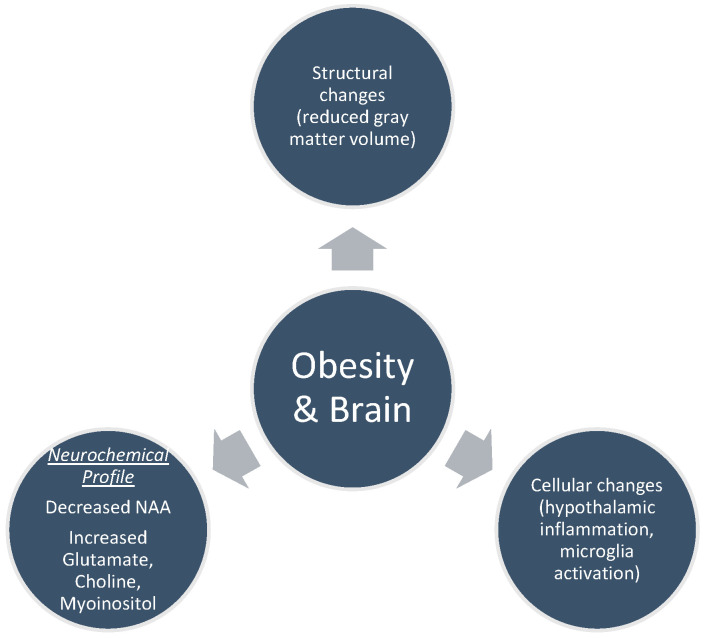
Obesity and brain function.

Obesity correlates with alterations in both the structure and function of the brain (Figure 2) [40,41]. These changes involve how the brain responds to external stimuli like food-related signals, taste, and scent, as well as alterations in its baseline activity, patterns of activation, and connectivity during different cognitive activities such as learning, impulsive decision making, inhibitory control, memory, and attention [42]. Brain imaging in animal studies reveals that diet-induced obesity results in morphological alterations of various cell types, including oligodendrocytes, microglia, astrocytes, and neurons [43]. Obese subjects, as shown by MRI scans, display elevated hypothalamic gliosis, which is corroborated by histological evidence revealing amplified microglia activation in individuals with a BMI exceeding 30 compared to those with a BMI below 25 [44].

### 4.1. Structural Changes

Obesity is associated with modifications in gray matter/white matter volume as well as white matter integrity [45]. Studies in adults show that certain inflammatory markers associated with obesity have been correlated with reduced volume of the brain, particularly affecting gray matter volume (GMV) over that of the white matter [46,47]. As the structure and function of the brain undergo continuous changes throughout development, especially in frontal regions, findings observed in adults may not be applicable to younger subjects. Consequently, it is crucial to conduct separate studies focusing on children and adolescents. Several studies indicate that children with overweight or obesity have lower GMV compared with their normal-BMI counterparts [48]; however, the existing evidence in youth is limited because it predominantly relies on small cross-sectional studies.

Few are also the studies that examined the relation between the dorsolateral prefrontal cortex (DLPFC) and the orbitofrontal cortex (OFC) and obesity in adolescents [49,50]. Subjects with overweight and obesity showed elevated activation of the prefrontal cortex (PFC) before meals and a smaller decrease in PFC activation after meals compared to normal-BMI subjects, while the OFC in subjects with obesity showed significant activation after meals compared to normal-BMI subjects [51].

### 4.2. Cellular Changes

Primarily based on studies involving diet-induced obesity in animals, specific brain regions, such as parts of the cerebral cortex or hippocampus, show distinct alterations in obese animals compared to lean ones. These changes include reduced spine density and disrupted myelin in neurons, along with an increased presence of activated microglia and reactive astrocytes characterized by enlarged soma size [52,53]. These cellular modifications most likely stem from the release of various proinflammatory molecules, such as saturated and monounsaturated fatty acids and cytokines (IL-6 and TNFα), into the bloodstream by the expanding adipose tissue. This release creates a hyperlipidemic, proinflammatory environment. Disrupting the functional and structural integrity of the blood–brain barrier results in an increased influx of lipids, proinflammatory substances, and immune cells into brain tissue, which fosters neuroinflammation [54]. As a consequence, microglia and astrocytes adopt a more proinflammatory and cytotoxic profile, compromising their roles in maintaining balance and supporting nerve cell growth. Both microglia and astrocytes heighten the production of proinflammatory cytokines and reactive oxygen species, which impairs mitochondrial function and causes damage to DNA, proteins, and cell membranes. This cascade of events leads to cell death in the surrounding environment, including neurons and oligodendrocytes [55].

In obesity and inflammatory states, lipid droplets build up in the cytoplasm of microglia, astrocytes, and neurons [56]. These droplets sequester excess lipids to prevent cell membrane disruption caused by lipid overload. Reactive oxygen species increase lipid production in neurons, which then transfer fatty acids to astrocytes. The lipid droplets within astrocytes absorb these fatty acids, leading to the production of proinflammatory factors, such as IL-6 and TNFα. Microglia also accumulate lipid droplets under inflammatory conditions, compromising their phagocytic function [57]. These observed cellular changes in the brain in obesity and inflammation need further investigation to understand their relationship with the observed volumetric and tissue magnetic resonance properties [43].

## 5. Magnetic Resonance Spectroscopy and Brain’s Neurochemical Profile

With the progress in neuroimaging, MRI techniques in animal models are a valuable tool to understand the effect of high-fat intake on the brain’s function and structure [58]. Magnetic resonance spectroscopy (MRS) provides information about the neurochemical composition of the brain by measuring key metabolites. Due to its relatively limited sensitivity, only small molecules present in millimolar concentrations are typically detectable in an in vivo MR spectrum. Signals from choline (Cho), creatine (Cr), and N-acetylaspartate (NAA) are usually observed in the normal brain at long echo times, while compounds such as lactate and alanine may be detectable in pathological conditions where their concentration increases. Conversely, at shorter echo times, additional compounds such as glutamate, myoinositol, and lipids become detectable [59]. The main brain metabolites detected through MRS encompass NAA, myoinositol, choline, creatine, and glutamate. Obesity can cause systemic inflammation, affecting multiple organs including the brain [60]. Furthermore, the highest predictive ability for diagnosing central precocious puberty in boys is achieved by the combination of aspartate, myoinositol, choline, and creatinine [61].

### 5.1. N-acetylaspartate (NAA)

N-acetylaspartate, an intracellular anionic solute, can accumulate in concentrations up to 10 mM or more. Its concentration is modified in various human brain disorders, being one of the most widely recognized neuronal biomarkers [62]. Being the brain’s second most prevalent metabolite, NAA is produced within neurons and exists in exceptionally high levels in the CNS [63], reaching detectable levels in neuronal tissue and not in other brain tissues, such as glial cells. Findings from various studies align to indicate swift rises in total NAA from prenatal stages through infancy, succeeded by gradual increments during childhood and adolescence [64]. The involvement of obesity in NAA metabolism shows that NAA levels decrease as BMI increases. Evidence suggests that NAA, a marker of neuronal integrity, decreases in overweight and obese subjects [65].

### 5.2. Myoinositol (MI)

Myoinositol is among the most plentiful metabolites found in the human brain, primarily situated within glial cells, where it serves as an osmolyte [66]. Myoinositol plays a role in cellular signaling and the synthesis of lipids [64]. Evidence suggests that MI levels are highest during the prenatal stage, gradually decreasing through infancy until stabilizing in early childhood. The significant drop in MI levels during the late prenatal and early postnatal period underscores its importance in brain development and implies a potential role in myelination [67]. Increased myoinositol concentrations in the brain have been found in conditions such as diabetes mellitus and Alzheimer’s disease [68]. Haley et al. showed that increased BMI in adults has a significant correlation with higher cerebral myoinositol levels, regardless of age and gender [69]. MI has been documented to decrease in hepatic encephalopathy [59].

### 5.3. Choline

Choline is recognized as playing an essential part in neurodevelopment, and it is considered to play a crucial role in maintaining the structural integrity of membranes and facilitating neurotransmission. Studies report that glial cells also exhibit high levels of choline. Choline possesses the capacity to modulate gene methylation and expression, thereby potentially influencing neuronal activity [70]. While humans produce choline in limited quantities, the majority typically rely on dietary sources to supplement endogenous choline production and prevent deficiency [71]. HFDs are frequently linked to cardiovascular disease, partly due to the breakdown of dietary choline by the gut microbiota into trimethylamine (TMA), which is absorbed in the intestines, contributing to the development of atherosclerosis. However, research indicates that a greater intake of choline has been correlated with improved neural processing in overweight/obese adults [72]. Obesity can influence the function of the cholinergic system, thereby potentially affecting cognitive abilities. The availability of choline and acetyl coenzyme A and the activity of the biosynthetic enzyme ChAT are essential factors in regulating the synthesis of acetylcholine (ACh). The synthesis of acetylcholine in cholinergic neurons is catalyzed by choline acetyltransferase (ChAT) [73]. Animal studies showed an elevated expression of choline acetyltransferase (ChAT) in obese rats [74].

### 5.4. Glutamate (Glu)

Glutamate acts as an excitatory neurotransmitter and plays metabolic roles in the Krebs cycle, the glutamate–glutamine cycle, nitrogen regulation, and the synthesis of gamma-amino-butyric acid (GABA). Glutamate is stored as glutamine in glial cells, and maintaining a harmonious interchange between these two neurochemicals is crucial for the optimal operation of brain cells. Advancements in magnetic resonance spectroscopy (MRS) have enabled the investigation of neuronal metabolism involving glutamate and GABA [75]. Glutamate levels have been demonstrated to be elevated in individuals with visceral obesity. Evidence suggests that circulating glutamate exhibits a strong correlation with visceral adipose tissue. This suggests glutamate’s potential as a screening tool for identifying visceral obesity and metabolic profile changes [76].

### 5.5. Creatine

Creatine is a marker of energy utilization, contributing to the cerebral energy supply [64]. In typical brain tissue, the creatine (Cr) peak observed in MRS results from a balance between contributions from free creatine (fCr) and phosphocreatine (PCr), each making up roughly half of the total. In the normal brain, creatine exhibits significant regional discrepancies, with levels generally lower in white matter compared to gray matter [59]. During the prenatal period and infancy, there is a continual increase in tCr levels, which stabilize by adolescence [64]. Furthermore, the NAA/Cr ratio is regarded as a marker of functional integrity, neuronal density, and overall brain activity. Neves et al. found that higher BMI z-scores are associated with lower NAA/Cr ratios in the hypothalamus [77].

## 6. Discussion

The hypothalamus, a part of central nervous system (CNS), contains important nuclei that serve as neuroendocrine cells. It plays a significant role in maintaining metabolic homeostasis [78], reacting to hormonal and neuronal signals, as well as nutrients from the bloodstream, and regulating food intake. Serving as a “metabolic sensor”, the hypothalamus becomes susceptible to excessive calorie intake and obesity-related increases in inflammatory molecules, such as cytokines and SFAs [79]. Overweight or obesity leads to the breakdown of the blood–brain barrier, allowing inflammatory substances to infiltrate brain tissue [80]. Diffusion-basis spectrum imaging restricted fraction (DBSIRF) in the hypothalamus is higher in overweight/obese children compared with normal-BMI children, reflecting neuroinflammation-related cellularity. This finding aligns with the neuroinflammatory pattern involving the recruitment, proliferation, and activation of astrocytes and microglia observed in this brain region in rodents exposed to HFDs. While this immune response may initially offer neuroprotection, chronic gliosis results in disrupted neuroinflammatory processes, affecting hypothalamic metabolic regulation. This disruption contributes to overeating, leptin and insulin resistance, and the onset of obesity. Prolonged neuroinflammation could also lead to axonal damage and neuronal loss [14]. Brain inflammation extends beyond the hypothalamic region and impacts adjacent areas of the brain as well.

HFDs are linked to the release of cytokines by non-neuronal cells in the hypothalamus, triggering metabolic inflammation. These cytokines then stimulate different inflammatory agents within the ARC nucleus of the hypothalamus, including inhibitors of κB kinase-β (IKKβ) and protein kinase C (PKC). The ARC nucleus is a central region of the hypothalamus, housing neurons associated with pro-opiomelanocortin (POMC). Interactions between non-neuronal cells and POMC neurons within the ARC nucleus result in a disruption of the balance between food intake and energy expenditure. While many connections are yet to be uncovered to fully comprehend HFD-induced metabolic inflammation within the ARC nucleus, it is evident that this inflammation impacts POMC neurons, leading to obesity [81]. Rodent studies have demonstrated decreases in the number of POMC cells following prolonged HFD consumption, along with elevations in indicators of apoptosis and neuronal stress within ARC nucleus neurons.

Childhood obesity represents a significant global public health concern. Obesity manifests as a condition characterized by chronic inflammation and elevated levels of insulin, lipids, and leptin. Changes in brain structures and architecture resulting from obesity strengthen the connection between obesity and central nervous system pathologies. Neuroinflammation resulting from obesity has been demonstrated to impact various regions of the brain, including the hippocampus, cortex, and amygdala. Obesity- and HFD-induced neuroinflammation can impact the hypothalamus, leading to various outcomes. Animal and human studies show that both a diet rich in trans-fats and obesity cause hypothalamic inflammation with subsequent alterations of the appetite-regulating hormones [82]. Additionally, obesity-induced hypertension is attributed to the direct enzyme activation in POMC neurons located in the mediobasal hypothalamus. This mechanism operates by activating the sympathetic nervous system [83].

While inflammation in peripheral tissues typically occurs over several weeks to months of HFD consumption in rodent studies [84,85], hypothalamic inflammatory markers become elevated within just 24 h of exposure to an HFD. Thaler J. et al. showed a roughly 25% decrease in the quantity of POMC cells in the ARC nucleus of mice after 8 months of exposure to an HFD, compared to those in the control group that were fed regular chow during the same period of time [86]. In addition, in rodents subjected to an HFD, the hypothalamus released proinflammatory cytokines (TNF-α, IL-1β), which then triggered apoptotic signaling in the hypothalamus [87]. Puig et al. analyzed diffusion tensor imaging (DTI) metrics in the hypothalamus of 24 middle-aged subjects with obesity and showed that DTI detects obesity-associated hypothalamic damage, which in turn is linked with inflammatory markers and poorer cognitive performance [88].

The initial method used to evaluate hypothalamic inflammation and gliosis involved establishing a T2 signal ratio based on standard techniques. In a study of 37 adults, a positive correlation was found between the MBH/amygdala T2 signal ratio and BMI. This correlation was specifically observed in the left hypothalamus [89]. In a cohort comprising 169 children aged 9–11 years, findings revealed a positive correlation between the MBH/amygdala T2 signal ratio and BMI z-score [90]. Additionally, children within the healthy weight range exhibited lower MBH/amygdala T2 signal ratios compared to those classified as overweight or obese. Increasing acknowledgment is being given to the connection between obesity and brain health. MRS provides information concerning alterations in brain metabolites that could potentially be associated with changes in both function and structure. Spectroscopy has the capacity to detect and monitor metabolic changes non-invasively. One- and two-dimensional spectroscopy techniques are essential tools for investigating and quantifying alterations in the significant brain metabolites. The development of the brain’s chemical composition, particularly the metabolites associated with different aspects of brain development, remains relatively understudied. Metabolic adaptations occur in the obese human brain, yet the underlying mechanisms have remained unknown. NAA, predominantly found in neurons, plays a role in facilitating communication between neurons and oligodendrocytes, as well as contributing to myelin synthesis by oligodendrocytes. Cr is engaged in cellular energy metabolism and storage, while Cho serves as an indicator of the structural integrity and turnover of cell membranes [91]. There is currently no evidence from research studies to establish a correlation between brain metabolites and CPP.

However, the above studies present a few limitations. Firstly, the presence of hypothalamic inflammation in boys with CPP has yet to be confirmed. Consequently, it is uncertain whether this phenomenon affects both genders equally or primarily impacts females. Secondly, the sequence of occurrence of disorders in the hypothalamus and CPP is ambiguous, leaving it unclear whether these phenomena stem from a shared pathological process or if hypothalamic inflammation autonomously initiates CPP.

## 7. Conclusions

The above data indicate that either acute hypothalamic inflammation due to short-term overnutrition or hypothalamic structural alterations owing to chronic obesity and/or inflammation may lead to disruption of the typically precise balance between caloric intake and energy expenditure. Certain observable changes in the “obese brain” that can be functionally demonstrated may revert with loss of weight. Meanwhile, the endurance of other abnormal patterns aligns with the idea that they might play a role in the pathophysiology of obesity [92]. Introducing interventions for the prevention and management of overweight and obesity could represent a key strategy in preventing precocious puberty. There are data that strongly advocate for interventions targeting families and social environments, prioritizing the promotion of reduced screen time and computer usage among children [93]. Persistent overweight and obesity during early childhood could potentially elevate the risk of CPP, particularly among girls. Implementing weight loss interventions may serve as a crucial strategy in preventing precocious puberty in children. The hypothalamic inflammation observed in mice experiencing diet-induced obesity could be reversed through the direct intracerebroventricular (ICV) administration of pure fatty acids, specifically omega-3 and omega-9. This intervention also results in a decrease in spontaneous food intake and a reduction in body mass gain [94]. However, the onset of puberty seems to be influenced by a high-fat diet irrespective of body weight. Given that several unhealthy food choices represent a significant concern in our society, and the prevalence of early and precocious puberty is rising, particularly among girls, it is crucial that we undertake research studies in children to investigate the impact of diet-induced hypothalamic inflammation on GnRH neuronal activation. Furthermore, there are no human data on hypothalamic inflammatory markers in girls with CPP. Therefore, it is essential to determine whether hypothalamic inflammation is evident in girls with or without obesity and early or precocious puberty. In addition, further studies are required to assess whether early or precocious puberty is associated with hypothalamic inflammation due to obesity and/or an HFD or other mechanisms. Finally, more studies are required to improve the assessment of hypothalamic inflammation via spectroscopy and fMRI or biochemical markers in girls with CPP. Exploring the association between overweight and/or obesity in early childhood and its potential role in increasing the risk of early or precocious puberty could also help prevent the development of early activation of the HPG axis.

## Data Availability

Not applicable.
